# Distinct and Overlapping Roles of Nipah Virus P Gene Products in Modulating the Human Endothelial Cell Antiviral Response

**DOI:** 10.1371/journal.pone.0047790

**Published:** 2012-10-19

**Authors:** Michael K. Lo, Mark E. Peeples, William J. Bellini, Stuart T. Nichol, Paul A. Rota, Christina F. Spiropoulou

**Affiliations:** 1 Centers for Disease Control & Prevention, Viral Special Pathogens Branch, Atlanta, Georgia, United States of America; 2 Centers for Disease Control and Prevention, Measles, Mumps, Rubella, and Herpes Virus Branch, Atlanta, Georgia, United States of America; 3 The Research Institute at the Nationwide Children's Hospital, Columbus, Ohio, United States of America; 4 The Ohio State University, Department of Pediatrics, Columbus, Ohio, United States of America; Commissariat a l'Energie Atomique(cea), France

## Abstract

Nipah virus (NiV) is a highly pathogenic zoonotic paramyxovirus that causes fatal encephalitis in up to 75% of infected humans. Like other paramyxoviruses, NiV employs co-transcriptional mRNA editing during transcription of the phosphoprotein (P) gene to generate additional mRNAs encoding the V and W proteins. The C protein is translated from the P mRNA, but in an alternative reading frame. There is evidence from both *in vitro* and *in vivo* studies to show that the P gene products play a role in NiV pathogenesis. We have developed a reverse genetic system to dissect the individual roles of the NiV P gene products in limiting the antiviral response in primary human microvascular lung endothelial cells, which represent important targets in human NiV infection. By characterizing growth curves and early antiviral responses against a number of recombinant NiVs with genetic modifications altering expression of the proteins encoded by the P gene, we observed that multiple elements encoded by the P gene have both distinct and overlapping roles in modulating virus replication as well as in limiting expression of antiviral mediators such as IFN-β, CXCL10, and CCL5. Our findings corroborate observations from *in vivo* hamster infection studies, and provide molecular insights into the attenuation and the histopathology observed in hamsters infected with C, V, and W-deficient NiVs. The results of this study also provide an opportunity to verify the results of earlier artificial plasmid expression studies in the context of authentic viral infection.

## Introduction

Nipah virus (NiV) is a highly pathogenic paramyxovirus in the genus *Henipavirus* of the subfamily *Paramyxovirinae* within the family *Paramyxoviridae* [1]. Fruit bats of the genus *Pteropus* are a natural reservoir for NiV [2], [3], [4], [5]. Humans are infected by exposure to infected fruit bats or material contaminated by infected bats; by intermediate hosts, like infected pigs; or by direct human-to-human contact [6], [7], [8], [9], [10]. The first known human NiV infections were detected during an outbreak of severe febrile encephalitis in peninsular Malaysia and Singapore from the fall of 1998 to the spring of 1999 [11]. NiV has subsequently been established as the cause of fatal human encephalitis in Bangladesh since 2001 almost yearly, and was detected in India in 2001 and 2007 [12], [13]. While the mortality rate during the initial Malaysian outbreak was ∼40%, the mortality rates in Bangladesh and India have consistently been closer to ∼75% [14]. In humans, NiV causes severe encephalitis characterized by systemic vasculitis and necrosis particularly in the central nervous system (CNS), as well as in the lung, heart, and kidney. Extensive infection of neurons, endothelial cells, and smooth muscle cells of blood vessels is characteristic of human NiV infections [15].

**Figure 1:**
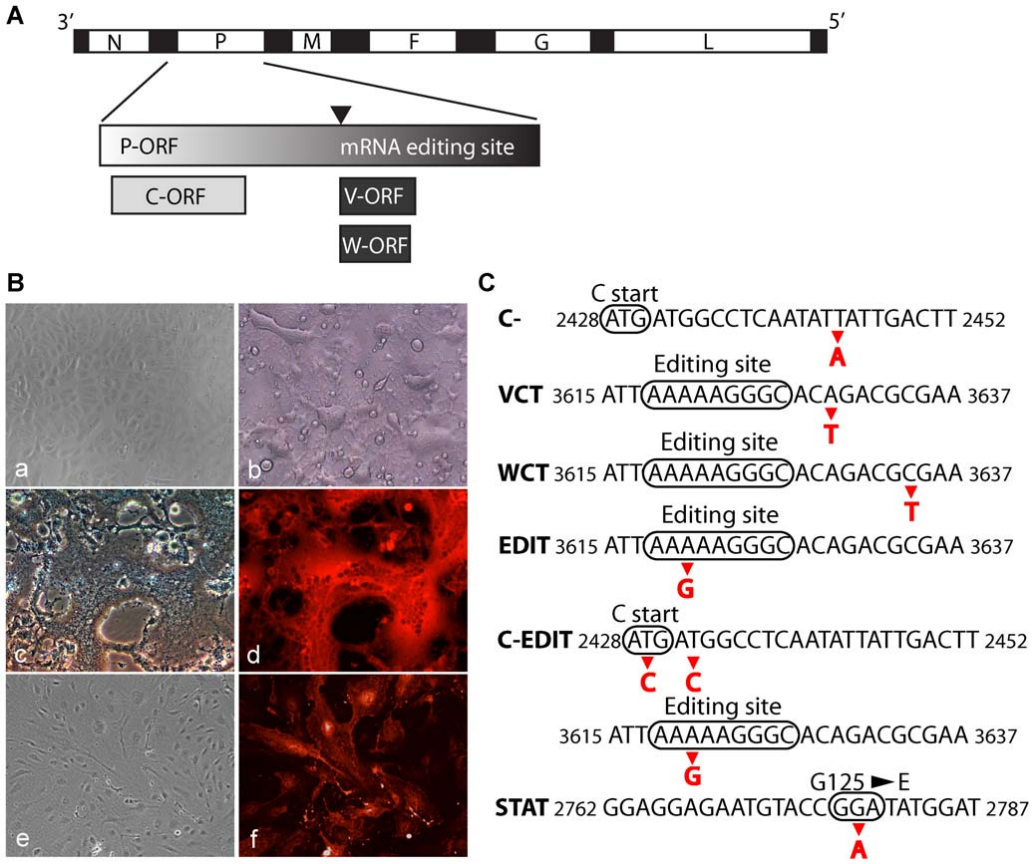
Nipah (NiV) P gene editing, and the establishment of a NiV reverse genetic system. (**A**) Schematic of Nipah (NiV) genome with the name of each gene indicated. Black segments represent non-coding regions of the virus genome, while the white regions indicate open reading frames (ORF). Inset magnifies the P gene and indicates the accessible ORFs in the NiV P gene due to mRNA editing (NiV V and W) and to a downstream alternative ORF (NiV C). Approximate location of the mRNA site is indicated by the black arrowhead. (**B**) Establishment of NiV reverse genetic system. Panel a: mock-infected Vero cells. Panel b: Wild-type recombinant NiV infection of Vero cells at 48 h post infection (PI) (MOI = 1). Panels c–d: light and fluorescence micrographs of Vero cells infected with recombinant red fluorescent (RED2AM) virus, 48 h PI (MOI = 1). Panels e–f: light and fluorescence micrographs of primary human microvascular lung endothelial cells (HMVEC-L) infected with RED2AM virus, 24 h PI (MOI = 2). (**C**) Generation of recombinant NiVs. Nucleotide changes (indicated by red arrowheads and letters) were incorporated into the NiV P gene to ablate expression of the C, V, and W ORFs, to alter the mRNA editing site, or to prevent STAT-1 binding in the shared N-terminal regions of the P, V, and W proteins. Numbers flanking the nucleotide sequences indicate plus-sense antigenomic position.

Like other paramyxoviruses, NiV employs co-transcriptional mRNA editing of the phosphoprotein (P) gene to generate additional mRNAs encoding the V and W proteins, and utilizes an alternative reading frame to generate the C protein [16], [17], [18], [19], [20], [21], [22] (Figure 1A). A number of studies using plasmid transfections of each NiV P gene product have indicated that all 4 protein products antagonize the cellular antiviral response to some extent. Individual expression of the C, V, and W proteins was able to restore replication of an interferon (IFN)-sensitive virus [23]. The N-terminal region shared by the NiV P, V, and W proteins prevents IFN signaling by blocking the phosphorylation of Signal Transduction Activator of Transcription 1 (STAT-1) [24], [25], [26]. The unique V protein C terminus counteracts IFN beta (IFN-β) induction by cytoplasmic RNA helicases mda-5 and RIG-I [27], [28], [29]. A nuclear localization signal in the unique C terminus of the NiV W protein enhanced its ability to antagonize interferon regulatory factor 3 (IRF-3)-dependent IFN-β induction [30].

A study which utilized recombinant NiV with specific mutations introduced suggested that the C protein was important for viral replication, and that the W protein was the primary inhibitor of STAT-1 activation in African green monkey (Vero E6) cells [31]. However, whereas nuclear localization of NiV W was confirmed in infected Vero and human neuroblastoma cells, pronounced cytoplasmic localization of NiV W was found in 3 distinct primary human endothelial cell types, corresponding with their respective abilities to generate a detectable antiviral response [21], [32]. Interestingly, an *in vivo* study of NiV infection of hamsters used recombinant mutant NiVs to indicate that the NiV V and C proteins serve as virulence factors; hamsters survived infection with NiVs lacking either the C protein or the unique C-terminal region of the V protein, but not with a W protein-deficient NiV [33]. The molecular mechanisms underlying this dramatic attenuation observed in the hamsters infected with V or C protein-deficient viruses are still unclear.

To clarify the respective roles of the NiV P gene products in virus replication and in counteracting the cellular antiviral response, we infected primary microvascular lung endothelial cells (HMVEC-L) with recombinant NiVs containing various mutations in the P gene. Our results indicate that multiple elements encoded by the P gene have both distinct and overlapping roles in modulating virus replication as well as in limiting the cellular antiviral response. Our findings corroborate observations from an *in vivo* hamster infection study, and provide molecular insights into the attenuation and the histopathology observed in hamsters infected with C, V, and W-deficient NiVs [33]. In establishing this reverse genetic system for NiV, we also expressed a non-viral reporter gene incorporated into the NiV M gene of our recombinant virus without the addition of a separate transcription unit into the genome.

## Results

### Initial rescue of recombinant NiVs

To initiate establishment of a NiV reverse genetic system, we were successful in rescuing the recombinant wild-type (WT) NiV, and also a recombinant red fluorescent reporter NiV (RED2AM) which allows rapid assessment of infection of different cell types (Figure 1B). The DsRed-Express (Clontech) red fluorescent protein was incorporated into the NiV matrix (M) gene in-frame with the M ORF; the FMDV 2A protease site was situated between the 2 ORFs to separate the translated proteins. This strategy precluded the requirement for an additional gene transcriptional unit to express non-viral genes. The RED2AM virus maintained expression of the DsRed-Express protein through at least 5 passages on Vero cells (the most we have passaged it so far), and was able to infect primary human microvascular lung endothelial cells (HMVEC-L) (Figure 1B). As done in earlier work, we also rescued recombinant NiVs deficient in either the C protein (C-), the unique V protein C terminus (VCT), or the unique W protein C terminus (WCT) (Figure 1C) [33]. We proceeded to rescue 3 additional mutant NiVs. We introduced a silent mutation into the P gene ORF within the RNA editing site to create a putative editing site mutant NiV (EDIT). By combining the C protein deficiency with the RNA editing site mutation, we generated a double mutant (C-EDIT) NiV. Lastly, we generated a point mutant (STAT) in the shared N-terminus of the P, V, and W proteins which ablated the ability of these proteins to bind Signal Transducer Activation of Transcription 1 (STAT-1) (Figure 1A, C) [34]. All rescued recombinant NiVs induced syncytia formation as seen in NiVs isolated from Malaysia (Figure 1B). We sequenced each rescued virus to confirm the respective engineered mutations and unique restriction sites.

### Characterization of recombinant NiVs in Vero cells

**Figure 2:**
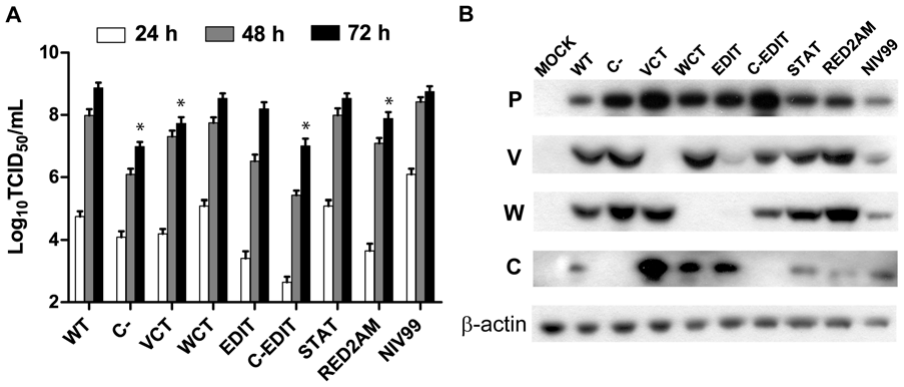
Characterization of recombinant NiVs in Vero cells. (**A**) Comparative viral growth curves. Vero cells were infected with each indicated recombinant NiV in triplicate wells at an MOI = 0.01. At the time points indicated, infected cell supernatants were serially diluted before being used to infect Vero cells to calculate log median tissue culture infectious dose per milliliter (Log_10_ TCID_50_/mL). Error bars indicate one standard deviation of the mean of triplicate infections. ANOVA with Dunnett's multiple comparison test was used to measure the statistical significance of differences in peak viral titer at 72 h PI when compared with WT recombinant virus. * p<0.001. (**B**) Western Blot analysis of Vero cell lysates infected with recombinant NiVs. Vero cells were infected at an MOI = 0.01. At ∼36 hours PI, infected cell lysates were harvested in NET-BSA buffer run on a 4–12% Bis-Tris SDS gel. Proteins were transferred onto PVDF membranes, blocked in 5% milk, and incubated with various antipeptide sera against NiV P gene products as indicated on the left hand side. Names of recombinant NiVs indicated above respective lanes. β-actin served as a loading control.

We used Vero cells to determine the growth curves of each rescued recombinant NiV (Figure 2A). The WT virus grew to almost identical peak titers as the initial Malaysian isolate (NIV99). The RED2AM virus grew to a slightly lower titer than WT (∼0.5 Log TCID_50_/mL). The WCT and STAT viruses grew to similar peak titers as WT, while the VCT and EDIT viruses grew to moderately lower titers than WT (∼0.5–1 Log TCID_50_/mL). The C- and C-EDIT viruses however grew to noticeably lower titers than WT (∼2 Log TCID_50_/mL). We consistently observed these differences across multiple growth curve experiments.

To confirm lack of viral protein expression from each of our recombinant mutant viruses, we performed Western blots on infected Vero cell lysates harvested at 36 hr post-infection (PI) using previously developed anti-peptide sera against the P, V, W, and C proteins (Figure 2B) [21]. The WT, RED2AM, and STAT mutant viruses expressed all 4 P gene protein products, while the C-, VCT, and WCT viruses respectively lacked expression of their intended individual mutagenized targets (Figure 2B). The EDIT virus had decreased levels of both V and W expression compared to the WT virus. The C-EDIT virus lacked expression of the C protein and had slightly lower levels of V and W compared to WT, but the decreased expression of these two proteins was not as pronounced as observed in the EDIT virus. The VCT, WCT, and EDIT viruses had increased expression of the C protein as compared with the WT. The C-, VCT, EDIT, and C-EDIT viruses also had relatively increased P protein expression compared to the WT (Figure 2B). We probed the lysates for β-actin as a cell lysate loading control for any significant differences observed in viral protein expression.

### Characterization of recombinant NiVs in HMVEC-L cells

**Figure 3:**
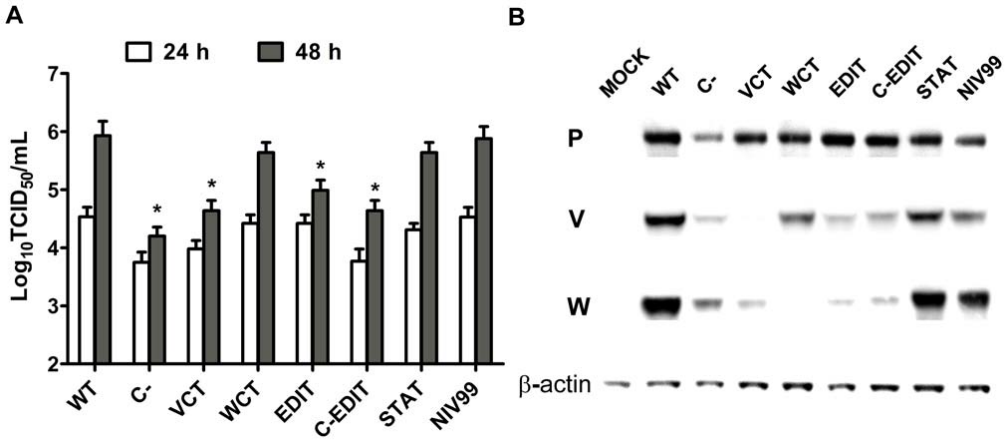
Characterization of recombinant NiVs in HMVEC-L cells. (**A**) Comparative viral growth curves. HMVEC-L cells were infected with each indicated recombinant NiV in triplicate wells at an MOI = 2 for 2 h before inoculum was removed and replenished with fresh media. At the time points indicated, infected cell supernatants were serially diluted before being used to infect Vero cells to calculate Log_10_TCID_50_/mL. Error bars indicate one standard deviation of the mean of triplicate infections. ANOVA with Dunnett's multiple comparison test was used to measure the statistical significance of differences in peak viral titer at 48 hours PI when compared with WT recombinant virus. * p<0.001. (**B**) Western blot analysis of P gene products from infected HMVEC-L cell lysates. HMVEC-L cells were infected with each indicated recombinant NiV at an MOI = 2 for 2 h before inoculum was replaced with fresh media. At 24 h PI, infected cell lysates were harvested in NET-BSA buffer and run on a 4–12% Bis-Tris SDS gel. Proteins were transferred onto PVDF membranes, blocked in 5% milk with TBS-T, and probed with mouse antipeptide sera against indicated proteins. NiV C protein could not be detected.

Pathological findings from the initial Malaysian outbreak of NiV indicated that along with neurons, endothelial cells of smaller arteries are major targets of NiV infection [15]. While all the recombinant viruses were able to generate syncytia in HMVEC-L cells, they were generally smaller than those observed in Vero cells (Figure 1B). We performed growth curves to determine the respective abilities of our recombinant NiVs to replicate in HMVEC-L cells (Figure 3A). Although the overall peak viral titers in HMVEC-L cells were noticeably lower than those seen from the Vero cell growth curves, we were still able to observe differences in growth kinetics among the viruses. As seen in the Vero cells, the WT virus grew to similar titers to NIV99 in HMVEC-L cells. The WCT and STAT viruses grew to similar peak titers as WT, while the EDIT virus had marginally lower peak titers (∼0.5 Log TCID_50_/mL). The C-, VCT, and C-EDIT viruses consistently had the lowest peak titers compared to the WT (∼1.0–1.5 Log TCID_50_/mL) across multiple growth curve experiments. Our previous observations indicated that peak viral titers for NiV infection were attained in HMVEC-L cells by 48 h, which precluded the use of later time points in this study [32].

We performed Western blots on infected HMVEC-L cell lysates taken at 24 h PI to determine the expression profile of P gene products for each recombinant virus. Our mouse polyclonal serum was unable to detect the C protein by Western Blot in any of the infected HMVEC lysates at 24 h PI, presumably due to lower levels of virus replication (data not shown). Despite lower overall levels of replication in HMVEC-L cells, we were nonetheless able to detect differences between the viruses in their respective expression of the P, V, and W proteins (Figure 3B). The C- virus had lower levels of all 3 proteins when compared to WT virus. We observed a significantly lower level of W protein expressed by the VCT virus while its P protein level was similar to WT. The EDIT virus expressed very little of either the V or W proteins, as observed in the Vero lysates, and had levels of P protein comparable to if not higher than WT. The C-EDIT virus expressed more P protein compared to WT, and lower levels of the V and W proteins. The WCT virus expressed marginally less P and V protein compared to WT, and the STAT virus had comparable levels of all 3 proteins to WT.

### Determination of RNA editing frequencies of recombinant P gene mutant NiVs

The differences in expression of NiV P gene products among our mutant recombinant viruses prompted us to determine the P gene mRNA editing frequencies of our recombinant viruses at 24 h PI in HMVEC-L cells (Table 1). The WT virus edited approximately 70% of its P gene mRNA transcripts, which was similar to findings from prior studies of NiV P gene mRNA editing [21], [22]. The C-, WCT, and STAT viruses also edited their respective transcripts at similar frequencies to WT. The VCT virus however only edited 36% of its transcripts, which is approximately half the frequency observed in the WT. Moreover, the EDIT and C-EDIT viruses were shown to edit only 12% of their respective P gene mRNA transcripts, which is effectively 5 fold less editing than the WT (Table 1). Taken as a whole, the RNA editing frequencies of these viruses observed in the HMVEC-Ls are consistent with the P gene protein expression profiles observed in both Vero and HMVEC-L Western blots (Figures 2B, 3B).

**Table 1:** NiV P gene mRNA editing frequencies in HMVEC-L cells (24 h post-infection).

Virus ID	Total clones	Edited (%)	Edited P	Edited V	Edited W	Unedited P
WT	41	29 (71)	3	14	12	12
C-	46	34 (74)	11	9	14	12
VCT	61	22 (36)	5	10	7	39
WCT	54	39 (72)	11	20	8	15
EDIT	90	11 (12)	2	5	4	79
C-EDIT	66	8 (12)	0	2	6	58
STAT	65	47 (72)	7	15	25	18
NIV99	50	35 (70)	10	17	8	15

### NiV P gene encoded elements limit expression of antiviral genes

**Figure 4:**
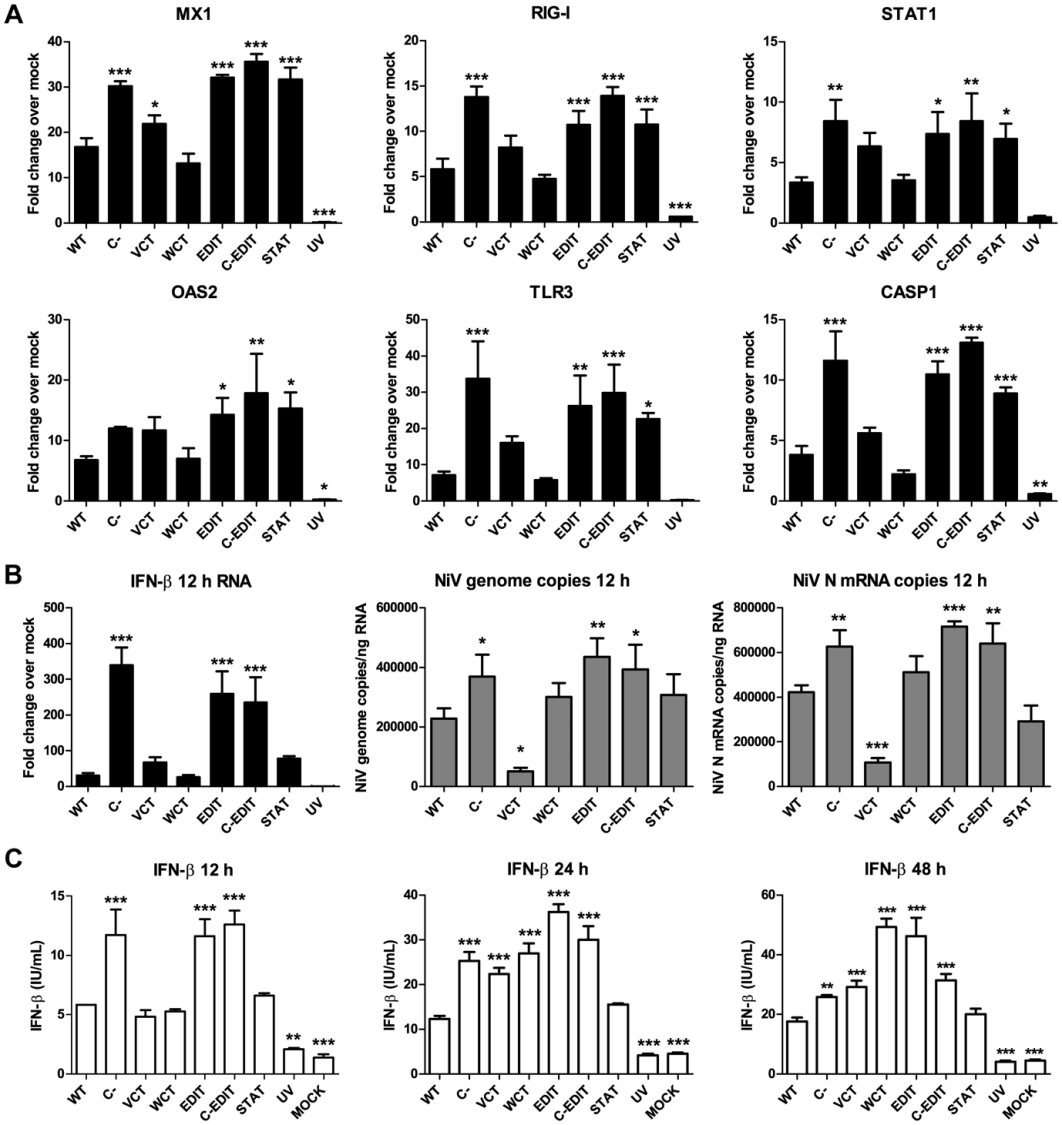
Multiple NiV P gene encoded elements contribute to limit the antiviral response. (**A**) NiV P gene encoded elements limit transcription of antiviral genes. HMVEC-L cells were infected with each recombinant NiV at an MOI = 5 and harvested at 12 h PI for total RNA extraction, reverse-transcription, and ensuing real-time PCR array. Levels of mRNA transcription of each antiviral gene indicated over mock were calculated by the delta delta Ct method (see materials and methods). (**B**) *Left panel*: Levels of IFN-β transcription induced by recombinant NiVs at 12 h PI (MOI = 5). Total RNA was extracted and reverse-transcribed as per the above to be used in an antiviral real-time PCR array. Levels of mRNA transcription of IFN-β over mock were calculated by the delta delta Ct method (see materials and methods). *Center panel*: NiV genome copy numbers from infected HMVEC-L cells at 12 h PI. Total RNA was extracted as mentioned above and reverse-transcribed using a primer against the 3′ leader sequence to only detect genome copies. Primers against the N gene were utilized to amplify viral genome. *Right panel*: NiV N gene mRNA copy numbers from infected HMVEC-L cells at 12 h post-infection. Total RNA was extracted as mentioned and reverse-transcribed using an oligo-dT primer to detect NiV N gene mRNA copies. Primers against the N gene were utilized to amplify N gene mRNA. (**C**) Induction of IFN-β secretion by mutant recombinant NiVs. HMVEC-L cells were infected with individual mutant NiVs at MOI = 5. At 12 h (left panel), 24 h (center panel), and 48 h (right panel) post-infection, infected cell supernatants were collected and were subject to IFN-β ELISA to measure levels of IFN-β induced by each mutant virus. Error bars for all panels indicate standard deviation of triplicate samples. ANOVA with Dunnett's multiple comparison test was used to measure the statistical significance of differences in viral replication and transcription, and IFN-β expression for each mutant compared with the WT virus. * p<0.05; ** p<0.01; *** p<0.001.

In order to determine the respective roles of the P gene products in counteracting the cellular antiviral response, we infected HMVEC-L cells with each recombinant virus at a high multiplicity of infection (MOI = 5) to ensure robust infection, and extracted total RNA from cell lysates at 12 h PI to evaluate the early antiviral transcriptional response. We evaluated transcriptional changes of 84 genes involved in the antiviral response, which included antiviral effectors such as Myxovirus resistance 1 (Mx1) and 2′–5′ oligoadenylate synthetase 2 (OAS2), pattern recognition receptors such as RNA helicase DDX58 (RIG-I) and Toll-like Receptor-3 (TLR-3), and signaling molecules such as Signal Transduction Activator of Transcription-1 (STAT1) and Caspase-1 (CASP1). While the WT virus induced detectably higher mRNA levels of these antiviral mediators over mock-infected levels, the C-, EDIT, C-EDIT, and STAT mutant viruses consistently induced significantly higher transcription levels of these genes (Figure 4A). The VCT virus also induced higher transcription levels of these antiviral genes, but the differences were less pronounced. Given that the VCT, EDIT, and C-EDIT viruses have significantly decreased levels of V and W proteins (Figure 3B, Table 1), the results suggest that the C protein and the STAT-1 binding region of the shared N-termini of the V and W proteins contribute to limiting the expression of these antiviral genes.

### Multiple NiV P gene encoded elements contribute to limiting IFN-β induction

Since the abovementioned antiviral genes are induced via type-1 interferon (IFN) signaling [35], [36], [37], [38], [39], [40], we also measured IFN-β expression induced by each virus at the transcriptional and translational levels. At 12 h PI, the C-, EDIT, and C-EDIT viruses induced significantly higher levels of IFN-β mRNA than WT, while the VCT, WCT, and STAT viruses induced similar levels to WT (Figure 4B, left panel). To investigate potential mechanisms of the increased induction of IFN-β by the C-, EDIT, and C-EDIT viruses, we measured the RNA copy numbers of NiV genome and NiV N gene mRNA transcripts by quantitative real-time PCR. Interestingly, the early induction of IFN-β transcription by the abovementioned mutant viruses corresponded with their respectively higher levels of genome replication and viral mRNA transcription (∼1.5 to 2 fold increase) as compared to WT (Figure 4B, center and right panels, respectively). The VCT virus however, had significantly lower copies of viral genomic and mRNA transcripts compared with WT (∼4 fold decrease), while the WCT and STAT mutants had similar levels of replication and transcription compared with WT. The differences observed between WT and the mutant viruses were reproducible over multiple experiments. RNA copy numbers were normalized according to levels of glyceraldehyde phosphate dehydrogenase (GAPDH) present per ng of total RNA.

At 12 h PI, the levels of IFN-β protein expression detected from infected cell supernatants corresponded with the increased IFN-β mRNA levels induced by each respective mutant virus (Figure 4C left panel). The C-, EDIT, and C-EDIT viruses each induced significantly higher levels of IFN-β in cell supernatants compared to WT induced levels, while the VCT, WCT, and STAT viruses induced levels comparable to WT (Figure 4C, left panel). By 24 h PI however, VCT and WCT viruses had also induced higher levels of IFN-β compared to WT (Figure 4C, center panel). At 48 h PI, the WCT and EDIT viruses had induced the highest peak levels of IFN-β, while increases observed for the C-, VCT, and C-EDIT viruses from the 24 to the 48 h time point were less pronounced (Figure 4C, center and right panels). The STAT virus induced similar levels of IFN-β when compared with WT at every time point we tested. With the exception of the STAT virus, the peak IFN-β levels induced by each mutant NiV corresponded to its respective peak viral titer (Figure 3B, Figure 4C right panel). These results indicate that the C protein and the shared N-termini of the V and W proteins have significant roles in limiting the early (12 h) induction of IFN-β during NiV infection, while the unique C-termini of the V and W proteins contribute to restricting later IFN-β expression (24 and 48 h).

### NiV P gene products inhibit the expression of antiviral chemokines

**Figure 5:**
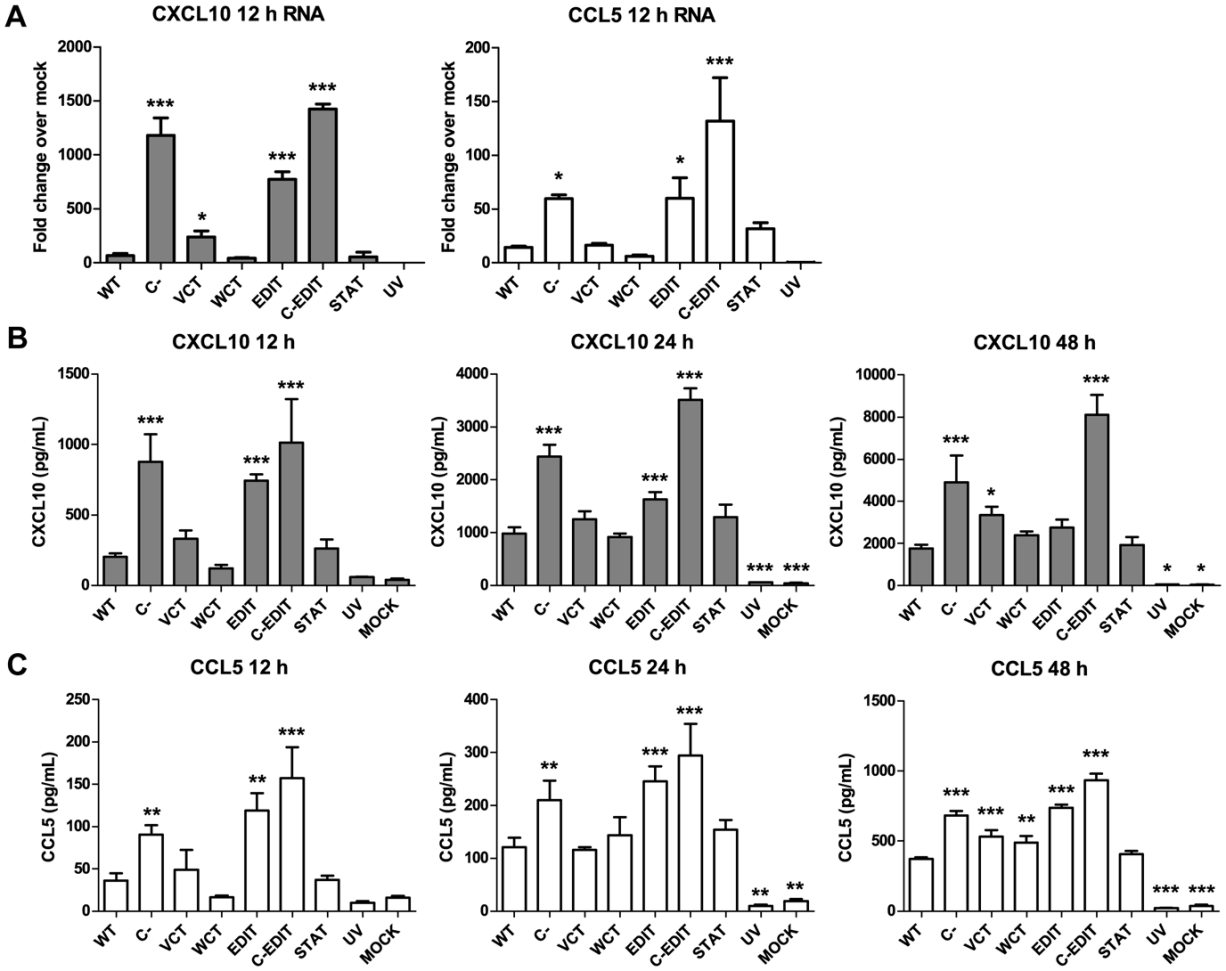
NiV P gene products inhibit the expression of antiviral chemokines. (**A**) RNA extracted from HMVEC-L cells infected at MOI = 5 with individual mutant NiVs at 12 h post-infection was reverse-transcribed into cDNA and used in an antiviral real-time PCR array to measure transcriptional induction of CXCL10 and CCL5. Antiviral chemokine mRNA transcription over mock levels was calculated by the delta delta Ct method (see materials and methods). Differential induction of (**B**) CXCL10 (gray bars) and (**C**) CCL5 (white bars) secretion by mutant NiVs. HMVEC-L cells were infected with individual mutant NiVs at MOI = 5 for 2 h before replacement of inoculum with fresh media. At 12 h (left panel), 24 h (center panel), and 48 h (right panel) PI, infected cell supernatants were collected and were subject to Luminex bead analysis to measure levels of CXCL10 and CCL5 induced by each mutant virus. Error bars indicate standard deviation of triplicate samples. ANOVA with Dunnett's multiple comparison test was used to measure the statistical significance of differences between levels of chemokine induced by each mutant compared with the WT virus. * p<0.05; ** p<0.01; *** p<0.001.

Our previous work demonstrated that NiV induced antiviral chemokine expression in several types of human microvascular endothelial cells [32]. In this study, we compared the abilities of each respective recombinant NiV P gene mutant virus to induce inflammatory protein 10 (IP-10/CXCL 10) and regulated and normal T cell expressed and secreted (RANTES/CCL5), both of which are inflammatory antiviral chemokines [41], [42], [43]. At 12 h PI, the C-, EDIT, and C-EDIT viruses each induced significantly higher mRNA and protein levels of CXCL10 and CCL5 expression than WT (Figure 5A, 5B left panels). At 24 h PI, the three abovementioned mutant viruses continued to induce higher levels of both CXCL10 and CCL5 in cell supernatants, although the level of CXCL10 induced by the EDIT virus was less pronounced than at 12 h (Figure 5B, 5C, center panels). At 48 h PI, only the C-, C-EDIT, and VCT viruses induced significantly higher levels of CXCL10 than WT, although the levels induced by the EDIT, and WCT viruses were detectably higher than WT (Figure 5B right panel). The C, C-EDIT, VCT, and WCT viruses induced higher levels of CCL5 than WT at 48 h PI (Figure 5C right panel). The C-EDIT virus consistently induced the highest levels of CXCL10 and CCL5 at every time point tested. In contrast, the STAT mutant virus consistently induced similar levels of both chemokines as WT at every time point tested (Figure 5B center and right panels). These results indicate that the C protein and the shared N terminus of the V and W proteins (but not the STAT-1 binding region) have a major role in controlling early antiviral chemokine responses, and that the respective C termini of the V and W proteins have lesser roles in controlling these responses in latter stages of infection. Ablating the C protein expression and reducing the expression of the V and W proteins as observed with the C-EDIT mutant had a synergistic effect in the induction of these antiviral chemokines at both the RNA and protein levels (Figures 5A, B, C). Taken as a whole, the results of this study indicate distinct and overlapping roles of the P gene products in modulating the antiviral response.

## Discussion

In establishing our NiV reverse genetic system, we developed a red fluorescent RED2AM reporter NiV to rapidly assess infection in different cell types. In contrast to a previously developed NiV reporter virus [44], we demonstrated the ability to co-express a heterologous reporter protein and a viral protein in the context of a single viral gene transcriptional unit. Precluding the use of an additional transcriptional unit to express reporter genes may better preserve the transcriptional gradient of viral mRNAs observed during paramyxoviral infection. Further characterization and optimization of the RED2AM virus will facilitate the study of early tissue replication sites in animal models as well as the development of antiviral assays. We used our reverse genetic system to generate a number of recombinant NiV P gene mutant viruses. By evaluating growth curves and antiviral responses mounted against each virus in HMVEC-L cells, we found that multiple P gene encoded elements have distinct but also overlapping roles in modulating both virus replication and the cellular antiviral response (Table 2 provides a summary of our findings).

**Table 2:** Summary of NiV P gene mutation phenotypes in HMVEC-L cells relative to WT.

Virus ID	RNA editing	Early replication	Peak titer	Early ISG	Early IFN-β	Late IFN-β	Early Chemokine	Late Chemokine
C-	** = **	**+**	**- -**	**++**	**++**	**+**	**++**	**++**
VCT	**-**	**- -**	**- -**	**+**	** = **	**+**	** = **	**+**
WCT	** = **	** = **	** = **	** = **	** = **	**++**	** = **	** = **
EDIT	**- -**	**+**	**-**	**++**	**++**	**++**	**++**	**+**
C-EDIT	**- -**	**+**	**- -**	**++**	**++**	**+**	**++**	**+++**
STAT	** = **	** = **	** = **	**++**	** = **	** = **	** = **	** = **

**+** increased compared to WT; **-** decreased compared to WT; ** = **approximately equivalent to WT.

The results of our study provide a clarifying lens through which prior plasmid expression studies should be interpreted. Although a previous study described the NiV C protein as having a lesser ability to antagonize the antiviral response compared with the V and W proteins [23], our C- virus consistently induced higher levels of IFN-β than the VCT and WCT viruses at 12 h PI (Figure 4B, C). Our results establish a major role for the C protein in limiting the early antiviral response. Our NiV C- mutant displayed similar characteristics to a C protein-deficient Measles virus (MeV) that induced translational inhibition and increased expression of IFN-β (Figure 3B, 4B, C) [45]. Similar to MeV C, NiV C may have multiple functions, serving both as a replication inhibitor as well as a direct inhibitor of the IFN-β response [46], [47], [48]. It is noteworthy that C protein-deficient NiVs had significantly impaired replication even in IFN-β-deficient Vero cells, implicating IFN-β-independent mechanisms of controlling viral replication (Figure 2A, 3A) [31], [33], [49]. Further studies into the mechanistic actions of NiV C are ongoing.

While our findings confirmed the respective abilities of the unique V and W protein C termini to antagonize IFN-β induction [27], [28], [29], [30], they also experimentally demonstrated a comparatively later mode of action for both V and W proteins than for the C protein (Figure 4C) [50]. We identified an important role for the unique V protein C terminus in establishing early viral replication (Figure 4B), and a lesser but detectable role in limiting antiviral chemokine expression (Figure 5B, C). The decrease in early viral replication observed for the VCT mutant corresponds with its inability to antagonize the activation of IFN-β by multiple RNA helicases such as retinoic acid-inducible gene 1 (RIG-I), melanoma differentiation-associated gene 5 (MDA5) and laboratory of genetics and physiology-2 (LGP2), and may indicate its failure to inhibit even earlier IFN-independent antiviral responses [51], [52]. While the WCT mutant induced significantly higher IFN-β levels later on during infection, its induction of chemokine expression was only marginally higher than WT (Figure 5B, C).

Results from this study also identified a crucial role for RNA editing in limiting the antiviral response by controlling early viral replication (Figure 4B), which corroborates evidence that the V and W proteins inhibit minigenome replication [48]. Severe reduction or complete ablation of viral P gene mRNA editing in several paramyxoviruses has been shown to reduce virus replication and pathogenicity [53], [54], [55], [56], [57]. For each of our mutant viruses which had decreased levels of P gene editing (EDIT, C-EDIT, VCT), we observed a significant induction of antiviral responses above WT-induced levels along with lower peak viral titers. Our results support the hypothesis that the shared N-termini of paramyxovirus P/V/W proteins may limit excessive viral replication by interacting with N protein, which would in turn limit IFN-β activation via cytoplasmic RNA helicases [58], [59], [60], [61], [62]. The 50% decrease observed in P gene mRNA editing for the VCT virus was an interesting albeit unexpected result. A potential explanation for this could be that the site in which the V ORF early stop codon was incorporated was just several nucleotides from the putative editing site, which may have negatively affected the RNA editing frequency. While mutagenesis studies of nucleotides 5′ to the editing site have been performed for several paramyxoviruses [63], [64], the incorporation of early stop codons for paramyxovirus V proteins however has not definitively been shown to alter RNA editing frequency [53], [54]. On the other hand, another possibility could be that certain residues in the unique C-terminus of the NiV V protein itself has a role in facilitating RNA editing, as it has been shown for Sendai virus [65]. If the shared N termini of V and W proteins act as inhibitors of viral replication, the decreased expression levels of the V and W proteins by the VCT virus (due to less editing) may have led to increased viral replication at later time points during infection (after 12 h, due to relatively more functional P protein), which would correspond with the delayed kinetics observed for its induction of IFN-β, CXCL10, and CCL5. The increased viral replication at later time points for the VCT virus could explain the modest reduction in peak viral titer despite significantly lower levels of early viral RNA transcription and replication. In light of these findings, future studies should explore the possibility of generating a different VCT mutant which does not have altered P gene mRNA editing frequencies. If it is possible to do so, it would help to clarify between the roles of the V protein N and C termini in controlling virus replication and the antiviral response.

We confirmed the ability of the STAT-1 binding region in the shared N termini of the P, V, and W proteins to inhibit type 1 IFN signaling [24], [25], [26]. Distinct from a prior reverse genetic study, the STAT virus used in this study was not generated in the background of a C- virus [31]. Despite carrying a non-conservative point mutation in the overlapping C ORF (Asn→Asp), the STAT virus had growth characteristics very similar to recombinant WT virus, and induced similar levels of IFN-β, CXCL10, and CCL5. Although the effects of the STAT-1 binding mutation was evident in the virus' reduced ability to block early antiviral gene transcription (Figure 4A), our results agree with the suggestion that type I IFN signaling has a minor role in suppressing NiV replication [33].

Most importantly, our findings corroborate observations from an *in vivo* hamster infection study; they also provide molecular insights regarding the attenuation and the histopathology observed in hamsters infected with C, V, and W-deficient NiVs (referred to as (C-), (V-), and (W-) in [33]). The significantly lower peak viral titers observed from HMVEC-L cells infected with the C- and VCT viruses correspond with the detection of little to none viral RNA detected in tissues of hamsters respectively infected with analogous C- and V- mutant NiVs [33]. The inherently higher levels of early genome replication and mRNA transcription observed for the C- virus (Figure 4B) may contribute to persistently low levels of viral RNA detected in tissues from C- virus-infected hamsters. The consistently elevated levels of antiviral chemokines induced by the C- virus throughout the course of HMVEC-L infection (Figure 5B, C) corresponds with the mild inflammation and infiltration observed in the lungs of hamsters infected with the C- virus [33]. Contrary to the conclusion that the attenuation of the C- virus in hamsters is largely independent of IFN antagonism, the robust early induction of IFN-β and antiviral gene expression by the C- virus in our study suggests that IFN may actually be a significant factor underlying the observed attenuation (Figure 4) [33]. While this manuscript was under review, an additional *in vivo* study of a C mutant NiV displayed a similar pattern of elevated inflammation and reduced viral RNA levels in hamster tissues, which further support our findings [66]. The role of IFN in the attenuation of the V- virus however is not as clear. While the VCT virus in our study induced comparable levels of IFN-β as the C- virus at later time points in our study, the significantly decreased ability of the VCT virus to sustain early viral transcription and replication may also be a significant factor corresponding to the absence of detectable viral RNA in V- virus-infected hamster tissues (Figure 4B, C) [33]. Indeed, several studies have implicated the C termini of paramyxovirus V proteins as elements required for pathogenesis [55], [56], [57], [67], [68], [69]. Our observation that the WCT virus induced the highest peak levels of IFN-β later during infection may explain the report of consistently lower levels of viral RNA (5 to 10-fold less) detected in hamster tissues infected with W- virus as compared to WT, despite their similar abilities to cause fatal disease [33].

A study which found CXCL10 expression in brains of deceased human NiV cases suggested that NiV encephalitis may be induced by the overexpression of CXCL10 [70]. While CXCL10 may indeed be expressed at high levels in the brains of fatal human NiV cases, the results of our study postulates a delicate balancing act in which the NiV C, V, and W proteins modulate both viral replication and the antiviral response to prevent overexpression of antiviral mediators such as CXCL10 and IFN-β which would likely be detrimental to virus replication.

In summary, our study has elucidated the actions of NiV P gene products in the context of primary microvascular endothelial cell infection, which not only provides a clearer perspective on prior plasmid expression studies, but also corroborating molecular insights into mechanisms of NiV pathogenesis in the hamster model. We have previously shown that primary endothelial cells respond distinctly to NiV infection compared to other immortalized cell types [32], [71]. It will be interesting to determine whether there are cell-type specific responses to NiV infection in different subsets of immune cells. Although one study suggested that NiV utilizes lymphocytes to disseminate systemically though an infected host [72], further studies are required to determine the nature of NiV interactions with immune cells which are crucial to mounting an effective antiviral response. Future experiments involving the infection of Pteropid bat cell lines with the P gene mutants developed in this study may delineate species-specific antiviral pathways in responding to different NiV mutants. Results from such experiments in turn could provide new insights into how bat innate immune responses contribute to apparent non-pathogenic henipavirus infection, as opposed to relatively high pathogenicity observed for their mammalian counterparts [73], [74], [75], [76], [77], [78], [79], [80], [81], [82], [83], [84].

## Materials and Methods

### Cells and viruses

BSRT7/5 cells [85] (a gift from Dr. Biao He) were maintained in Dulbecco's Modified Eagle's Medium (DMEM) high glucose (Invitrogen, Grand Island, NY, USA), supplemented with 7.5% fetal bovine serum (FBS), 10% tryptose phosphate broth (BD Biosciences, Franklin Lakes, NJ, USA), 100 U/mL penicillin, 100 µg/mL streptomycin, 2 mM L-glutamine, and 0.4 mg/mL Geneticin (Gibco, Grand Island, NY, USA). African green monkey (Vero and Vero-E6) cells were maintained in DMEM supplemented with 7.5% FBS, 100 U/mL penicillin, and 100 µg/mL streptomycin. Primary human microvascular lung endothelial cells (HMVEC-L; previously referred to as HULEC in [32]) were maintained in EGM-2 MV medium (Lonza, Walkersville, MD, USA) with 5% FBS supplemented with hydrocortisone, human epidermal growth factor, vascular endothelial growth factor, human fibroblast growth factor, fibroblast growth factor basic, R3 insulin-like growth factor 1, ascorbic acid, heparin, and gentamicin/amphotericin-B as provided in the manufacturer's EGM-2 Bullet Kit.

### Plasmids, sequencing, and construction of recombinant NiVs

RNA was extracted from NiV-infected Vero-E6 cells infected with NIV99, the first isolate of NiV from a 1999 Malaysian human case (accession #AF212302) [11]. All live NiV infections were performed in biosafety level (BSL) 4 containment facilities at the Centers for Disease Control and Prevention (CDC; Atlanta, GA, USA). Viral RNA was isolated from cell lysates for reverse-transcription to cDNA [86]. A modified version of the low copy number plasmid pBR322 was used as a backbone for the NiV infectious clone [87]. The majority of the sequence encoding ampicillin resistance was excised in the modified pBR322 using the NotI and ApaI restriction sites. An artificial DNA following elements (5′ to 3′ in the order listed) was generated from 8 overlapping oligomers of roughly 100 nt each were assembled by PCR to produce the ‘backbone’ construct containing the template for the following elements is this order: the Not I restriction site, the T7 promoter (followed by ‘GGG’ to optimize transcription), a 5′ hammerhead ribozyme, the 5′ NiV antigenomic leader, the beginning portion of the N gene, a polylinker with restriction sites *Asi* SI, *Mlu* I, *Sbf* I, *Sac* II, *Fse* I, *Age* I, *Avr* II, and *Asc* I, part of the 3′ antigenomic non-coding region of the L gene, the 3′ antigenomic trailer, a hepatitis delta virus ribozyme, 2 T7 terminator sequences, and an *Apa* I restriction site. Individual genes from NiV were PCR-amplified from cDNA reverse-transcribed from viral RNA using Superscript II and Platinum Taq High Fidelity kits (Invitrogen). To facilitate downstream cloning procedures, unique restriction enzyme (RE) sites were included in each primer sequence so that each open reading frame was flanked by two unique restriction sites, one in the antigenomic 5′ non-coding region (NCR) of that gene and one in the 5′ NCR of the preceding gene. All RT-PCR products were TA-cloned (Invitrogen) and their sequences confirmed to match the genomic sequence of NiV before inserting them into the backbone to generate the full-length NiV antigenomic cDNA clone. The backbone had been constructed so that the rule of six would be maintained once all the NiV genes were inserted [16], [88], [89]. The resulting plasmid with all the incorporated NiV genes, pNiV-WT, was transformed into DH5-alpha (Invitrogen) *E.* coli and was selected on tetracycline (35 µg/mL) (Sigma). The unique RE sites that we inserted in the antigenomic sense 5′ NCR of the N gene (nt 78–95) and in the antigenomic sense 3′ NCR of the L gene (nt 18155–18173) do not alter the motifs analogous to the (GNNNNN)_3_ motif in Sendai virus and the repeated (CGNNNN)_3_ motif in SV5 (PIV5) [90], [91], [92]. Due to its ∼7 kb length, the L gene was amplified in 2 fragments of 3 kb and 4 kb, with a natural internal RE site and modified flanking RE sites.

Site-directed mutagenesis of the P gene was performed by overlapping PCR using primers carrying the specific mutations. The C protein-deficient NiV mutant (pNiV-C-) was generated by incorporating an early stop codon in the C gene open reading frame (ORF) at antigenomic sense position 2447. Mutant NiVs deficient in the unique C terminus of the V protein (pNiV-VCT) or W protein (pNiV-WCT) were also generated by incorporating an early stop codon in their ORFs at antigenomic sense positions 3629 and 3634, respectively. The NiV editing site mutant (pNiV-EDIT) was generated by replacing an adenine with a guanine residue at antigenomic sense position 3620, which resulted in a silent mutation in the P gene ORF. We also generated a NiV containing both the C ORF and the editing site mutations (pNiV-C-EDIT). The editing site mutation of this construct was performed as mentioned for pNiV-EDIT. However, the C ORF was ablated by replacing the 2 thymine residues with 2 cytosines at antigenomic sense positions 2429 and 2432, resulting in silent mutations in the corresponding P ORF. The STAT-1 binding mutant was generated by replacing a guanosine residue with an adenosine residue at antigenome position 2779, which results in the G125E mutation reported in a prior study [34].

Following PCR amplification and purification of the P gene regions containing the appropriate P gene mutants, the fragments were digested with MluI and SbfI and cloned directly into the pNiV-WT plasmid digested with the same REs. For the pNiV-Red2AM reporter infectious clone, the DsRed-Express ORF (Clontech Laboratories Inc., Mountain View, CA, USA) was incorporated into the NiV infectious clone using 4 PCR amplification steps. First, an endogenous SbfI site in the dsRed ORF was removed by site-directed PCR mutagenesis. Next, the antigenomic 5′ NCR of the NiV M gene was incorporated at the 5′ end of the dsRed ORF, while the foot-and-mouth disease virus (FMDV) 2A protease cleavage sequence [93] was incorporated in-frame at the 3′ end of the dsRed ORF, in place of the original dsRed stop codon. Thirdly, the FMDV 2A protease cleavage site was also incorporated in-frame with the complete NiV M ORF, the M gene 3′ NCR, and part of the F gene 5′ NCR by PCR amplification. Finally, we used overlapping PCR with forward primer for the M gene antigenomic 5′ NCR and a reverse primer for the F gene antigenomic 5′ NCR to combine the PCR products from the second and third steps. The resultant PCR product was cloned into a TA vector and transformed into *E. coli*, which underwent ampicillin selection (100 µg/mL) on luria broth plates pre-treated with 150 µg of X-gal (Promega Corp, Madison, WI, USA) for both antibiotic and blue-white screening. Plasmid DNA was extracted from the white colonies selected in Luria broth with ampicillin, and was sequenced to confirm the presence and accurate amplification of the dsRed-2A-M ORF fragment. This fragment was then digested with SbfI and SacII and cloned into the pNiV-WT clone pre-digested with the same REs. The construct was transformed into *E. coli* and selected with tetracycline as above. The total genomic length of the Red2AM NiV was 18996 nt. Sequences of the primers used for all cloning steps are available upon request. To generate helper plasmids for virus rescue, the N gene (AsiSI, MluI), P gene (MluI, SbfI), and L gene (AgeI, AscI) fragments were cloned into a modified pCAGGS expression vector using these RE sites [94].

### Rescue of recombinant NiVs and purification of virus supernatants

LT-1 transfection reagent (Mirus Bio, Madison, WI, USA) was used to transfect BSRT7/5 cells in a 6-well plate format. pCAGGS helper plasmids expressing the N, P, and L genes, and a plasmid encoding the recombinant full-length antigenomic sequence of either the wild-type (WT) or mutant NiV, were co-transfected into these cells. The N∶P∶L∶genome plasmid ratio used was 1.25∶0.8∶0.4∶7.5 µg. At 48 h post-transfection, ∼10^5^ Vero cells were added directly to each well of BSRT7/5 cells. Cytopathic effect (CPE) due to NiV replication was generally observed by day 6–10 post-transfection. Supernatants from wells displaying CPE were transferred onto confluent monolayers of Vero cells in larger T-75 or T-162 flasks to grow a larger stock of virus. Supernatants and lysates of infected cells were collected and frozen at -70°C. Upon thawing, they were centrifuged at low speed (500 *g*) to pellet cellular debris, and the supernatant containing live virus was centrifuged through Amicon Ultra-15 centrifugal 100 kDa filter units (Millipore, Billerica, MA, USA) to significantly reduce the amounts of cytokines or chemokines potentially present in the supernatant. The concentrated supernatant remaining on the filter (∼250 µL) was resuspended in the original volume using Opti-MEM. The supernatant was then aliquoted, and serially diluted ten-fold to infect 3–4×104 Vero cells/well in a 96-well plate to determine the tissue culture infective dose 50 (TCID50)/mL. TCID50 was calculated using the Reed and Muench method [95].

### Immunoblotting

Western blots were performed as described previously [21]. Briefly, ∼1×10^5^ Vero cells/well of a 12-well plate, or 2×10^5^ HMVEC-L/well of a 6-well plate, were infected with a particular recombinant NiV clone at an MOI of either 0.1 (Vero) or 2 (HMVEC-L). The cells were harvested 36 h (Vero) or 24 h (HMVEC-L) later in 200 µL of NET-BSA buffer (150 mM NaCl; 5 mM EDTA; 50 mM Tris-HCL, pH 7.4; and 1 mg/mL bovine serum albumin), and irradiated in a cobalt-60 gamma irradiator with 2×10^6^ rad. Approximately 10 µL of cell lysate from each sample was run on a denaturing 4–12% SDS-PAGE gel (Novex, Grand Island, NY, USA). Proteins were transferred from the gel onto polyvinilidene fluoride (PVDF) membranes using the iBlot device (Invitrogen), and were blocked overnight in TBS-T (200 mM NaCl; 50 mM Tris-HCl, pH 7.4; 0.5% Tween) with 5% skim milk. PVDF membranes were incubated with polyclonal mouse antisera against P, C, V, or W [21] diluted in TBS-T with 5% milk (working dilution:1∶500 for P, C, V; 1∶1000 for W) for 1 h at room temperature, and washed 3 times in TBS-T. The membranes were then incubated with a goat anti-mouse IgG conjugated to horseradish peroxidase (HRP; 1∶10,000 dilution; Sigma-Aldrich) for 1 h at room temperature, washed 3 times in TBS-T, incubated with ECL reagent (Promega) for 1 min, and exposed to film for development. For sample loading controls, the blots were stripped with Reblot Plus Strong Antibody Stripping Solution (Chemicon, Billerica, MA, USA), blocked with TBS-T with 5% milk for 1 h, and washed 3 times in TBS-T. The blots were then incubated with a monoclonal rabbit anti-human β-actin antibody conjugated to HRP (Sigma-Aldrich; 1∶20,000 dilution) for 30 min, washed 3 times in TBS-T, incubated with ECL reagent (Promega), and exposed to film.

### Viral growth kinetics

Vero cells were infected with each recombinant NiV clone at MOI = 0.01. Viral inocula were removed 2 h post-infection (PI), and replaced with growth medium. Viral supernatants were collected 24, 48, and 72 h PI, and serially diluted ten-fold to infect 3–4×104 Vero cells in a 96-well plate to determine the TCID50/mL as above. HMVEC-L were inoculated with recombinant NiVs at MOI = 2 for 2 h, then given growth medium. Viral supernatants collected at 24 and 48 h PI, were serially diluted ten-fold to determine TCID50/mL as above.

### RT-PCR and cloning and sequencing of P gene mRNA transcripts

For cloning the editing sites, RNA was extracted from lysates of infected cells using Direct-zol RNA miniprep kit (Zymo Research Corp, Orange, CA, USA), and 8 µL of total RNA from each sample was used in an RT reaction using oligo dT_20_ primers (Invitrogen). PCR of the entire NiV P gene ORF was performed as previously described [96], except that the PCR cycling conditions were 94°C for 1 min 30 s, followed by 40 cycles of 94°C for 25 s, 50°C for 30 s, and 68°C for 2 min 45 s. The reaction was then incubated at 68°C for an additional 5 min. PCR products were visualized using agarose gel electrophoresis and purified using the QIAquick PCR purification kit (Qiagen). The primers used to amplify the entire P gene ORF, including the editing site, were NiV P1 (forward): 5′-ATGGATAAATTGGAACTAGTC-3′, and NiV P18 (reverse): 5′-TCAAATATTACCGTCAATGATG-3′. Purified PCR products were cloned into PCR2.1 using the Topo-TA Cloning Kit (Invitrogen) following the manufacturer's instructions. White colonies were picked and grown in 200 µL of LB in a 96-well format, and plasmid DNA was isolated using a MiniPrep Kit (EdgeBIO, Gaithersburg, MD, USA). At least 40 colonies containing the P gene amplicon of each individual viral clone were sequenced using a cycle sequencing reaction with BigDye 1.1 fluorescent dye terminators (Applied Biosystems, Carlsbad, CA, USA), and the reaction products were analyzed using ABI 3100 automatic sequencers (Applied Biosystems). The primers used for sequencing the editing sites were NiV P8 (forward): 5′- TGAGTGCTCTGGATCGGAAGA-3′, and NiV P13 (reverse): 5′-AATGATCTGCGTGATAATTCA-3′.

### Antiviral PCR assays

Quantitative PCR arrays (Qiagen) were used to determine up- or downregulation, relative to mock-infected cells, of a select panel of 84 antiviral genes in HMVEC-L infected with either recombinant WT or mutant NiVs. Each infection was performed in triplicate. For each sample, cDNA was synthesized from 0.5 to 1.0 µg of RNA using an RT^2^ first-strand kit (Qiagen). Arrays were run on an ABI 7500 PCR system using RT^2^ SYBR Green/ROX PCR master mix according to the manufacturer's instructions (Qiagen). Fold change over mock for each gene was calculated for each set of triplicate HMVEC-L infections using the threshold cycle (ΔΔ*C*_T_) method and normalized to the average values for 5 housekeeping genes (β2 microglobulin, hypoxanthine, ribosomal protein L13a, glyceraldehyde-3-phosphate dehydrogenase (GAPDH), and β-actin) (Qiagen). Four-fold increase in gene transcription over mock levels with p values of <0.05 was considered significant.

### Real-time PCR for determining NiV genome and mRNA copies

To determine NiV genome or N gene mRNA copy numbers, we used the NIP3END primer 5′-ACCAAACAAGGGAGAATATGGATAC-3′, and an oligo-dT_20_ primer (Invitrogen), respectively, to reverse transcribe total RNA extracted from HMVEC-L infected with various recombinant NiVs. RT was conducted using the Superscript III First Strand Synthesis kit (Invitrogen) according to manufacturer's protocols. Resulting cDNA was diluted 1∶5, and 2.5 µL of cDNA per sample was used in a 25 µL (total volume) RT^2^ SYBR Green/ROX real-time PCR using primers adapted from a real-time Taqman RT-PCR previously described [6]. To measure levels of GAPDH present in each sample, 2.5 µL of diluted cDNA was used in a separate SYBR Green real-time PCR using primers for GADPH [32]. Synthetic NiV N RNA standards of known quantity (∼10^9^ copies/µL) were reverse-transcribed using primer NIPN9 5′-TCACACATCAGCTCTGACGAA-3′ in a 20 µL reaction, and diluted 1∶5. The diluted cDNA (2.5 µL) was used to generate 10-fold dilutions for a standard curve to determine NiV N gene copy number [6]. We reverse-transcribed a specified amount of total RNA extracted from mock-infected HMVEC-L, and generated serial 10-fold dilutions of the resulting cDNA (diluted 1∶5) to create a standard curve plotting the total amount (ng) of RNA v. Ct values of GAPDH for each respective dilution. Based on this curve, we used the amount of GAPDH present in each sample to normalize the NiV genome or mRNA copy number results to the total amount of RNA in each sample.

### Interferon-β (IFN-β) ELISA

Assays were performed as described in [32]. Briefly, 100 µL of gamma-irradiated supernatants from NiV-infected cells were added to each well of a 96-well microplate pre-coated with affinity-purified polyclonal antibody to human IFN-β, along with 50 µL of horseradish peroxidase labeled-antibody. The reactions were incubated for 2 h on a plate shaker. After 3 washes, 100 µL of substrate was added to each well, and reactions were incubated at room temperature for 30 min before 100 µL of reaction-stopping solution was added to each well. The absorbance of the reaction mixture in each well was read at 450 nm, with the reference wavelength set to 630 nm.

### Luminex® multiplex cytokine bead assay

Assays were performed as described in [32]. 50 µL of gamma-irradiated supernatant per sample was used in Luminex cytokine bead assays according to manufacturer's protocols (Invitrogen). Briefly, supernatants were incubated for 2 h in a 96-well filter plate with a mixture of beads conjugated to antibodies specific for inflammatory protein 10 (IP-10/CXCL 10) and RANTES (CCL5). After 2 washes, the beads were incubated for 1 h with biotinylated detector antibodies specific for different epitopes of the aforementioned cytokines and chemokines. After 2 more washes, the beads were incubated with streptavidin conjugated with phycoerythrin (PE) for 30 min. After 3 washes, the beads were resuspended in 100 µL of wash buffer, and the fluorescence signal of the PE was read in the xMap® System using a Luminex® 200 IS cytometer (Bio-Rad, Hercules, CA, USA). Cytokine levels were measured using cytokine standards provided with the bead kits (Invitrogen).

### Statistical analyses

GraphPad Prism; GraphPad Software, Inc.). Analysis of variance (ANOVA) with Dunnett's multiple comparison test was used to determine statistical significance when comparing either peak viral titers, levels of protein, or RNA expression in HMVEC-L infected with WT recombinant NiV to those in cells infected with recombinant mutant NiV. p values<0.05 were considered significant.
